# Relationship between plasma cell-free DNA changes and lysyl oxidase during the treatment and prognosis of canine transmissible venereal tumors

**DOI:** 10.1186/s12917-022-03173-z

**Published:** 2022-02-21

**Authors:** Mona Mohamadzaheri, Hadi Cheraghi, Darioush Shirani, Ali Hatamkhani

**Affiliations:** 1 Department of Clinical Sciences, Faculty of Veterinary Medicine, Razi University, Kermanshah, Iran; 2 Department of Small Animal Internal Medicine, Faculty of Veterinary Medicine, University of Tehran, Tehran, Iran; 3 Oncology Service, Nikan Pet Hospital, Tehran, Iran

**Keywords:** Canine, Transmissible venereal tumor, Liquid biopsies, Cell-free DNA, Lysyl oxidase

## Abstract

**Background:**

Transmissible venereal tumors (TVT) are a wide range of canine tumors for which there are no effective markers to monitor the therapeutic response in real-time. Circulating biomarkers can be valuable in early cancer diagnosis and prognosis. Accordingly, this study aimed to investigate the significance of the cell-free DNA (cfDNA) and cfDNA integrity index to monitor the response of TVTs to vincristine and compare them with lysyl oxidase activity. Plasma and sera were collected from fifteen male dogs within four weeks before drug administration. The analytical method was mainly based on the quantitative polymerase chain reaction (qPCR) technique for short and long cfDNAs and lysyl oxidase activity was measured in serum.

**Results:**

The results of the cfDNA integrity index showed a significant (*p* < *0.05*) difference in the baseline concentration compared to the second and third weeks (with cut-off values of 1.118 and 93.33% specificity). The cfDNA integrity index increased over time due to the reduction of short cfDNAs in the first week after treatment. Lysyl oxidase activity increased during the fourth week (*p* < *0.001*), but there were no significant differences in the other weeks compared to the baseline. The ROC analysis of lysyl oxidase revealed high sensitivity (100%) and specificity (90%) on the second and third weeks compared to the baseline. Multivariate analysis between cfDNA integrity index and lysyl oxidase showed significant correlation (*p* < *0.05*) only in baseline results.

**Conclusions:**

Overall, short cfDNA, the cfDNA integrity index, and lysyl oxidase activity can be proposed as diagnostic biomarkers and putative prognostic candidates in TVT patients. These biomarkers can be combined with cytology to quickly diagnose TVT.

**Supplementary Information:**

The online version contains supplementary material available at 10.1186/s12917-022-03173-z.

## Background

The transmissible venereal tumor (TVT), also known as infectious sarcoma, sticker tumor, or transmissible lymphosarcoma, was recorded in all continents during the twentieth century [1, 2]. They are naturally occurring tumors transmitted between animals during copulation through viable tumor cells. Tumor cells primarily affect external genitalia and occasionally the internal genitalia, although some exceptions of additional engaged sites have been observed. The masses tend to bleed easily due to extensive ulceration of the epithelial surface lining [3].

Dogs with TVT experience pain and hemorrhages and exhibit usually cauliflower-like and red-colored serosanguinous discharge in the external genitalia. Hemorrhagic discharge is strongly associated with mucosal membrane-based TVTs, which can engage genitalia and oral and nasal cavities [3, 4]. The host’s immune system plays a vital role in inhibiting tumor growth and metastasis in TVTs. Younger dogs or dogs with compromised immune systems show higher tendencies for metastasis [5]. The tumor analysis is primarily based on a complete physical exam and a cytological analysis. Several treatments, including surgery, radiotherapy, immunotherapy, and chemotherapy, are implemented to treat TVTs [6]. Nonetheless, chemotherapy is regarded as the best and most practical approach for TVT treatment. In that regard. Vincristine sulfate is the primary solution for many patients [7, 8].

Liquid biopsy can assist a couple of programs throughout the continuum of canine most cancers care, which includes screening (for early detection) in excessive-threat populations, resources withinside the diagnosis, selection of targeted therapies, and tracking for most cancers recurrence or remedy reaction through serial checking out; and guarantees to bring the power of precision oncology to veterinary exercise thru an easy blood draw that does not require adjustments to the clinical routine [9]. Finally, a liquid biopsy check overlaying cancer-related regions of the genome with excessive homology among puppies and human beings can allow the identity of somatic changes in puppies with clinically actionable human homologs. Such insights received from liquid biopsy-primarily based totally genomic profiling might be used to hurry the adoption of targeted human most cancers therapeutics for the remedy of dog most cancers [10].

Cell-free DNA (cfDNA) has been suggested as a promising tumor marker. However, its level is also elevated in various non-malignant disorders. Therefore, more specific approaches, such as DNA integrity measurement, have been proposed [11]. Previous studies show that circulating biomarkers are advantageous over tissue biopsies due to the higher concentration availability in invasive procedures and the accessibility of withdrawing frequent samples in a period [9, 12]. Furthermore, cfDNA does not originate only from tumor cells [13]. It also originates from the tumor microenvironment and other non-cancer cells (e.g., endothelial or immune cells) from various body parts [14]. It seems to be the case that all cells can and are likely to release cell-specific DNA into the extracellular environment continuously. Therefore, diagnosis may be sufficient to look only at apoptosis-derived cfDNA originating from cancer cells. However, to better estimate tumor dynamics, mutation load, progression or assess the efficacy of treatment, the best approach may be to determine the proportion of aberrant vs wild-type DNA, including all forms of cfDNA [10].

The extracellular matrix provides essential structural and biochemical support to cancer cells. LOX is a secreted copper-dependent amine oxidase family member that plays a fundamental role in extracellular matrix remodeling and maturation [15, 16]. It is best known for initiating the crosslinking of collagen and elastin, stabilizing fibrous deposits, and enhancing cancer cell proliferation, metastasis, and angiogenesis. However, mature active LOX and LOX-PP play opposing roles in cancer progression. LOX-PP has been associated with tumor suppressor functions. The actions of LOX-PP have been proved to inhibit cell transformation, proliferation, and growth and induce apoptosis in various tumor cells [16, 17].

This study is a qualitative and quantitative assessment of cfDNA fragments and the cfDNA integrity index in canine transmissible venereal tumors (CTVTs) to examine their potential as diagnostic/prognostic markers and monitor therapeutic response in real-time. This study uses a comprehensive analysis of serum LOX and investigates its relationship with a CTVTs prognosis to assess its eligibility as a liquid prognostic biomarker.

## Results

### Circulating cfDNA in plasma and cfDNA integrity index

**Fig. 1:**
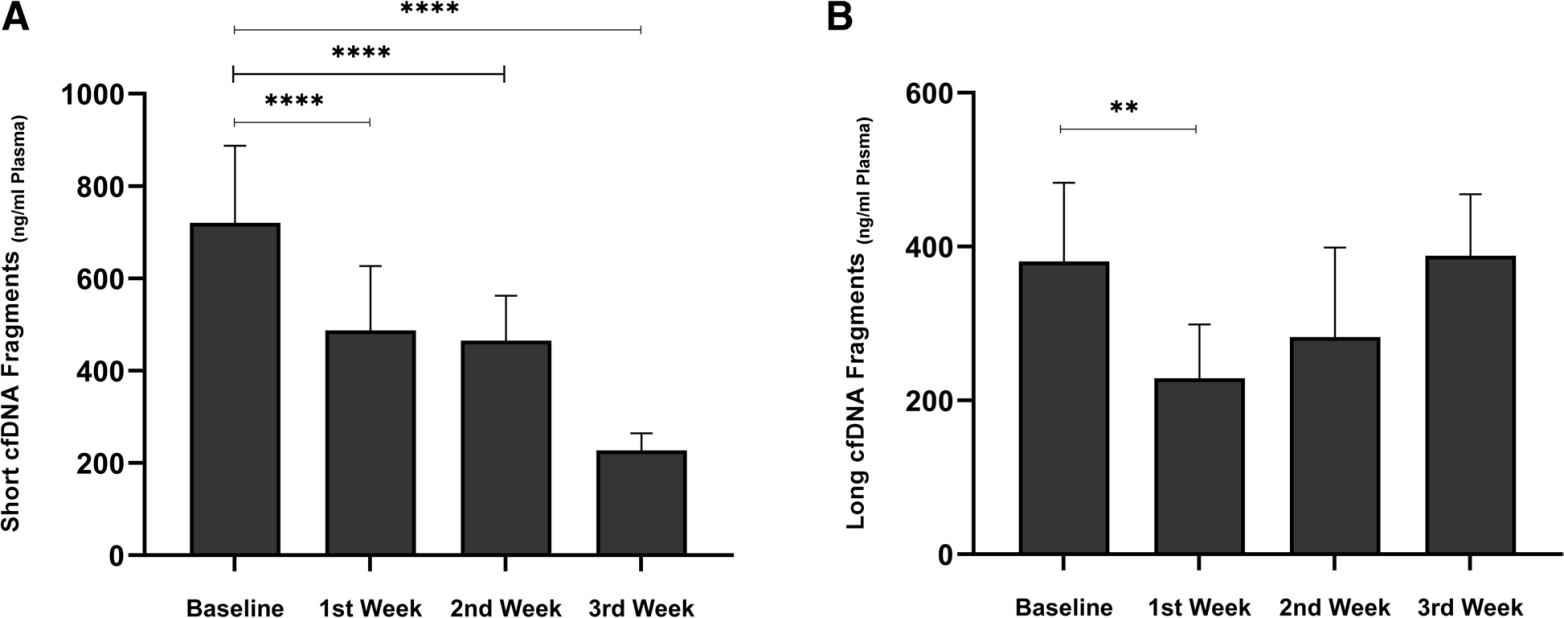
**A** Results for Short (Line-99) and **B** Long (Line-218) of plasma circulating cfDNA concentrations in dogs with four weeks of treatment. **P < 0.01 and ****P < 0.0001 vs. Baseline

Circulating cfDNA in plasma of dogs with TVT was assessed for short and long cfDNA fragments concentrations by real-time PCR, and then the integrity index was calculated (long/short) each week of treatment. When analyzing short cfDNA values from neoplastic dogs between weeks of treatment, a significant outlier was found (Fig. 1-A), which presented short fragments = 720.02 ± 167.49 ng/ml plasma (Mean ± SD) before prescribing vincristine and 487.38 ± 139.80, 465.11 ± 97.50, 227.17 ± 37.28 ng/ml for the first to the third week, respectively. Long cfDNA amount (*n* = 15) after treatment in the first week was significantly lower than neoplastic dogs before treatment; however, comparing results of long cfDNA in other weeks with baseline showed an insignificant relationship (Fig. 1-B). The long fragments result of the baseline compared to the last weeks of treatment were as follows: 380.89 ± 102.34, 229.02 ± 69.74, 282.23 ± 116.55, and 388.06 ± 80.35, respectively.

**Fig. 2:**
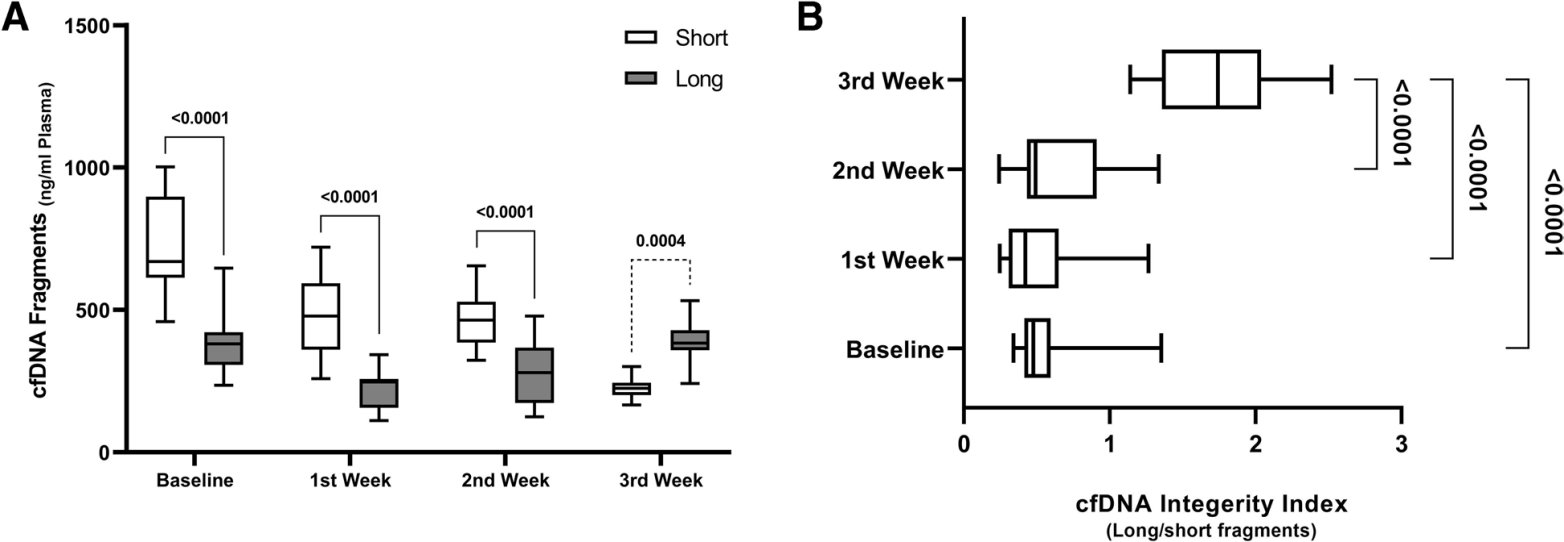
**A** Box-plot of short and long cfDNA for each week was shown. **B** Box plots with whiskers of comparing cfDNA integrity Index (Long/Short) of the effect of Vincristine treatment of the Dogs in the fourth week. Each box indicates the 25^th^ and 75^th^ percentiles. The horizontal line inside the box indicates the median, and the whiskers indicate the extreme measured values

Figure 2-A shows the differentiation between short and long cfDNA each week. Comparison of short and long results in subjects at all weeks of treatment revealed significant changes in short and long fragments concentration. Also, compared the results of the last weeks of treatment indicated that the number of long cfDNAs increased compared to the previous weeks.

Pre- and post-Integrity of circulating cfDNA integrity index results were shown in Fig. 2-B. A higher DNA Integrity Index was seen in the third week compared to other weeks (*p* < *0.0001*). Comparison of the DNA Integrity Index in other groups did not show any significant change (*p* > *0.05*).

### Lysyl oxidase activity

**Fig. 3:**
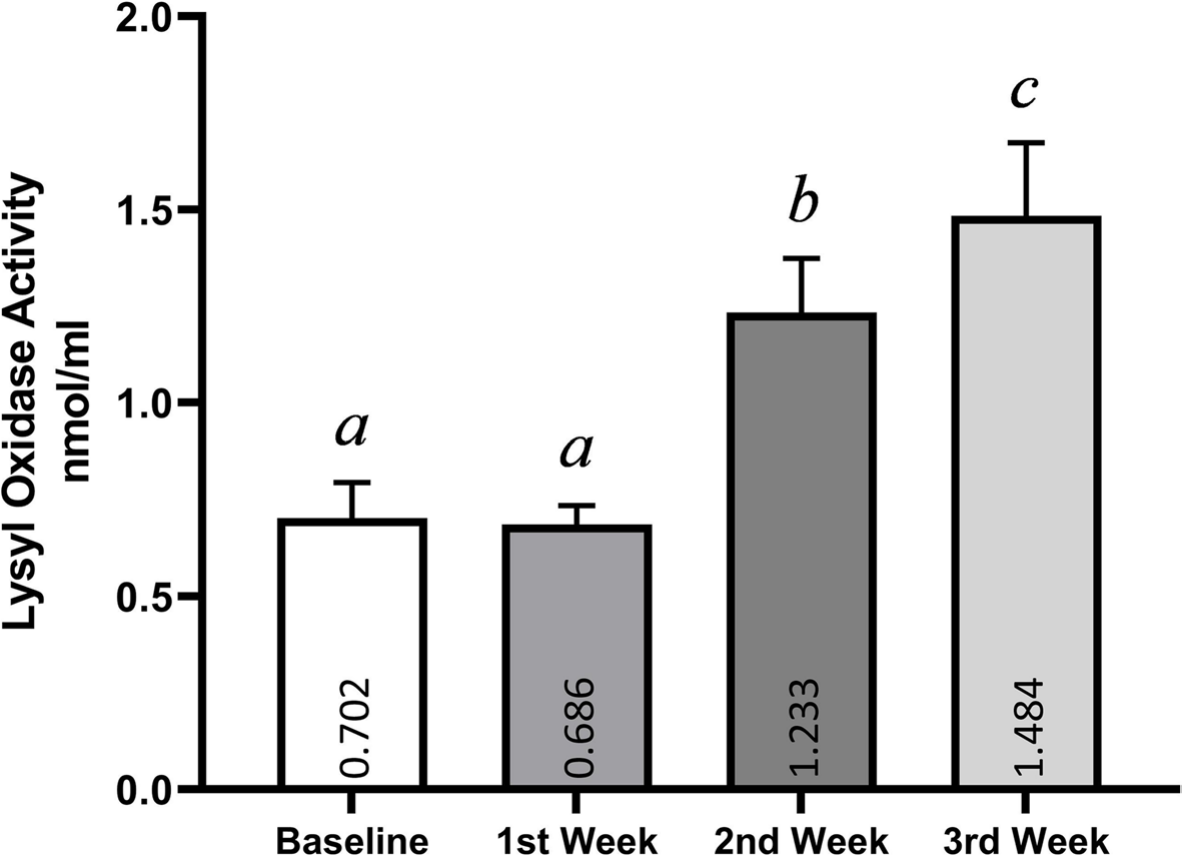
Lysyl oxidase activity for each week of Vincristine treatment. Data are expressed as Mean ± SD (Lysyl oxidase activity is expressed as nmol/ml). Values with non-identical letters (a, b and c) are significantly different (ANOVA, *p* < *0.05*)

Figure 3 shows the LOX activity of dogs with TVT in four weeks. As shown, LOX activity was higher in the second and third weeks. The highest amount (1.431 nmol/ml) of activity is related to the third week after treatment, while in the first week, there is no difference (*p* > *0.05*).

### ROC curves analysis for cfDNA integrity index

**Fig. 4:**
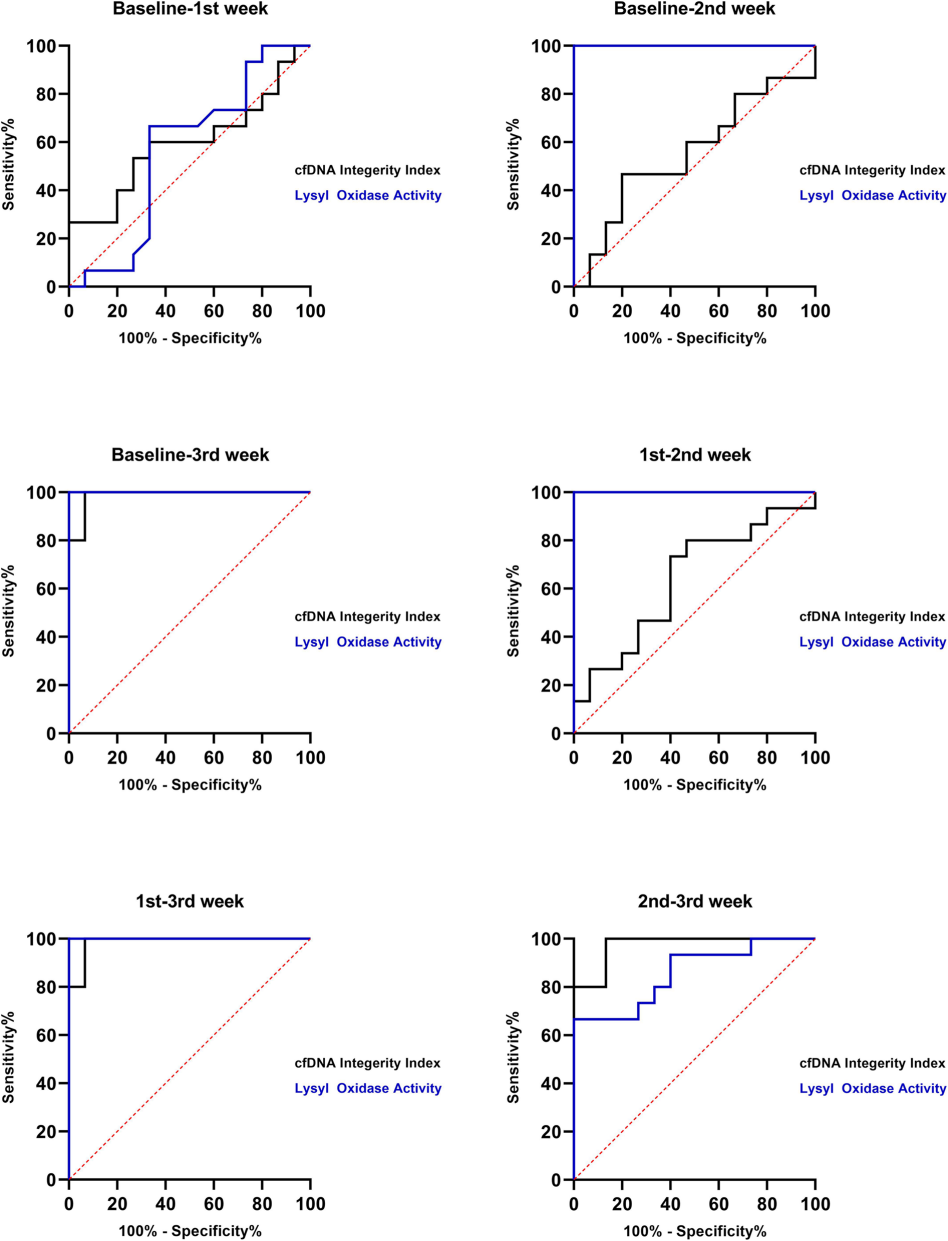
ROC curve of cfDNA integrity index Line 218/99 (Black lines) and Lysyl oxidase activity (Blue lines) in dogs with cytological diagnosis of TVT within the treatment

ROC curves were plotted to determine the cfDNA integrity index and discriminate the effect of treatments each week. The best cut-off result of ROC curves related to the comparison of the Baseline with the third week was 1.118 with 100% diagnostic sensitivity, 93.33% specificity, 99.66% positive predictive value, and 70.18% negative predictive value. In addition, the results of the ROC curve of cfDNA integrity evaluations of the other weeks together are shown in Fig. 4. The area under the curve was respectively 0.5956 for Baseline-1st week in canine with TVT, and 0.5556 and 0.9867 for Baseline-2nd and third weeks, respectively.

### The cut-point, sensitivity and specificity of cfDNA integrity and LOX activity

These observations were expanded by calculating sensitivity and specificity data (Table 1) obtained by ROC curves using 95% confidence intervals (Fig. 4). The cut-off point was higher than 0.5985 for Baseline-3rd week, with 55.56% AUC. Otherwise, the cut-off point of 1.118 (AUC: 0.93) was obtained for Baseline-3^rd^ week, providing high sensitivity levels with 100% AUC. There was strong sensitivity and specificity in cases before treatment and the third week (100% sensitivity, 93.33% specificity), consistent with the cfDNA Integrity Index. Poorer sensitivity results were seen in the first, second, and Baseline-1st weeks (26,67% sensitivity) (Table 1).

**Table 1:** Cut-off points, sensitivity, specificity and area under the curve (AUC) of ROC for cfDNA Integrity Index and LOX activity during treatment of canine Transmissible venereal tumor

	**Baseline-1**^**st**^** week**	**Baseline-2**^**nd**^** week**	**Baseline-3**^**rd**^** week**	**1**^**st**^** week-2**^**nd**^** week**	**1**^**st**^** week-3**^**rd**^** week**	**2**^**nd**^** week-3**^**rd**^** week**
**cfDNA integrity index**						
Sensitivity %	26.67	46.67	100.00	26.67	100.00	80.00
Specificity %	93.33	80.00	93.33	93.33	93.33	86.67
Cut off point	< 0.3485	> 0.5985	> 1.118	> 0.9070	> 1.022	> 1.329
AUC of ROC %	59.56	55.56	93.33	63.33	98.67	97.33
**LOX activity**						
Sensitivity %	60.00	100.00	100.00	100.00	100.00	62.50
Specificity %	70.00	90.00	90.00	90.00	90.00	87.50
Cut off point	< 0.6960	> 0.8648	> 0.8648	> 0.7396	> 0.7396	> 1.332
AUC of ROC %	58	100	100	100	100	81

Table 1 shows the ROC analysis of LOX enzymes activity in addition to cfDNA results. The cut-off point lower than 0.6960 for Baseline-1st week with 58% AUC for LOX activity revealed that the case had disease or inflammation. It could also be used as a marker to select the best treatment. A cut-off point of 0.8648 was obtained for comparing the baseline with two other weeks. There was strong sensitivity and specificity in cases Baseline-3^rd^ week, 1^st^-2^nd^, and 1^st^-3^rd^ weeks (100% sensitivity, 90% specificity), which is consistent with the LOX activity in serum of animals with TVT.

### ROC curves analysis for LOX activity

ROC curve analysis was also used to investigate the diagnostic performance of the LOX marker under study by comparing it with vincristine during treatment. Among the weeks of treatment, except for the Baseline-1^st^ week (the ROC was 0.6960), the rest of the weeks have acceptable sensitivity and specificity for using this marker in deciding for improvement or treatment process (Fig. 4).

### Correlation and multivariate analysis to determine of cfDNA integrity index and LOX activity

We evaluated the cfDNA integrity and LOX activity every week of treatment in the TVT dogs through correlation analysis (Table 2). CfDNA Data shown that 1st week (*r* = 0. 3541, *p* = *0. 1954*), 2nd week (*r* = -0.1051, *p* = *0.7094*) and 3rd week (*r* = -0.0023, *p* = *0.9933*) were not significantly correlated with LOX activity. But the remarkable thing about these results is that if the alpha is considered less than 0.05, the results show that the comparison between baseline will be significant (*p* = *0.0329*), which may be applied in the clinic or decided to continue treatment. Also, Table 2 presents multivariable regression analysis in TVT patients, for the associations between cfDNA integrity levels and the LOX activity in univariable analysis which there was only independent determinant for the plasma level of cfDNA integrity index with serum LOX activity before treatment.

**Table 2:** Relationship between the cfDNA integrity and LOX activity each week of treatment

	***Pearson's analysis***		***Multivariate regression analysis***			***P‐value***
	***r***	***95% confidence interval***	***R squared***	***β***	***t‐value***	
**Baseline**	-0.5521	-0.07991 to -0.1455	0.3852	-1.684	2.387	**0.0329**
**1**^**st**^** week**	0.3541	-0.1043 to 0.6884	0.1254	1.959	1.365	0.1954
**2**^**nd**^** week**	-0.1051	-0.5229 to 0.3535	0.0110	-0.2576	0.3809	0.7094
**3**^**rd**^** week**	-0.0023	-0.4440 to 0.4402	0.0001	-0.0052	0.0085	0.9933

## Discussion

Assays performed on neoplasm surrogate samples are engaging in the main because of the restricted invasiveness of sample collection. CfDNA determination might represent an inexpensive thanks to impacting on an attainable diagnostic, prognostic and observance tool in the medical specialty [14]. The rise of cfDNA has been reported in various diseases particularly inflammation situations, and it's been found also in human patients with various forms of cancer [18]. As yet, current cfDNA in blood has been thought about as a promising biomarker in various human tumors and, today, cfDNA fragmentation diagrammatic by the integrity index is studied for its ability to discriminate cancer patients from healthy [19]. Few knowledge are accessible for the canine neoplasms [20, 21]. During this study, we have a tendency to evaluate the extent of Line-99 and 218 cfDNA fragments by qPCR of the two amplicons with completely different lengths (short and long), within the plasma of Transmissible venereal tumor dogs.

By tracking the cfDNA concentration and cfDNA integrity index at several weeks in 15 Dogs with TVT, we found that cfDNA parameters correlated well with the clinical stage and tended to increase during or before periods of disease progression, suggesting comparability in monitoring the clinical stage, such that our statistics indicated that short cfDNA lower in a time-dependent manner, which reaches minimal concentration in the third week of remedy. Dogs with a cfDNA of higher than 1.118 value had a 93.33% probability of being dealt with due to the cfDNA integrity index. Circulating cfDNA are short fragmented DNA [22] that can be detectable in serum or plasma in order to be purified, quantified, and ultimately specially amplified via way of means of polymerase chain reaction (PCR) [23]. On the other hand, way to an upward push in cell death leading to an extended quantity of DNA fragments diffusing into a move in which acute ailment tactics had notably better elevations in brief cfDNA concentrations than continual ones [24]. Several studies decided on the patient-particular mutations diagnosed within the tumor tissues for cfDNA evaluation of plasma and proved the employment for postoperative surveillance with unique sequencing technology in animals [25–27] which the consequences from those research evaluated that analyses of mutations in cfDNA changed into strongly associated with ailment severity in a clinical setting. In agreement with formerly published data specifically in humans, in our study, the neoplastic puppies earlier than treatment confirmed a better quantity of each short and long cfDNA fragment than treatment weeks. Only a few studies [12, 28, 29], compared circulating DNA in non-neoplastic with neoplastic dogs (lymphoma, mammary tumors, and different cancers) and discovered comparable results.

Serum cfDNA integrity index (Long/Short cfDNA) is a potential molecular biomarker in diagnosis and prognosis of cancers which may be a beneficial indicator of disease status or discriminate most cancers from healthy Patients for physicians and veterinarians [12, 30, 31]. Increased necrosis inside the tumor led to noticeably fragmented DNA copies released into circulation, so Index ought to be lower in cancers [32]. Our Data indicated that this index sharply increased after three weeks of treatment in assessment with other weeks (*p* < *0.0001*), consequently this increase (decrease DNA fragmentation) turned into determined to be good after an anticancer treatment and the endurance of such modifications have been related to accurate prognosis [33]. Similar outcomes have been acquired in Dogs with mammary tumors in evaluation with healthful puppies [12]. That look affords evidence that cfDNA integrity index will be a diagnostic marker in puppies carrying mammary nodules suggesting that its ability application in early diagnostic procedures ought to be similarly investigated. Contrary to our results and the study noted above, a look at turned into performed on Feline diffuse iris melanoma especially which indicates cfDNA concentration and integrity evaluation found out no significant variations among the cats with iris melanoma and Healthy group [27]. The comparable cfDNA integrity outcomes have been Located in dogs with lymphoma or leukemia, hemangiosarcoma, and remote metastasis; cfDNA stages correlated properly with a medical stage and tended to increase in the course of or earlier than intervals of disease progression, suggesting ability efficacy of cfDNA for the detection of the remote. However, it additionally increases some other problems that cfDNA integrity won't be taken as a biomarker especially for some tumors however be more appropriate for treatment surveillance or prognosis in dog tumors [21, 26].

Moreover, we discovered a higher AUC value of cfDNA integrity in Baseline-third week results, it'd be rash to conclude cfDNA integrity is now no longer the suitable biomarker for the first weeks of treatment until it ought to be evaluated with a short cfDNA level. Nevertheless, we additionally executed ROC curve evaluation for every week and it'd be impractical to apply cfDNA integrity alone to the assessment of response to treatment in the first week of TVT. A value lower than the cutoff value (1.118) turned into taken into consideration awful prognosis, and smaller values have seemed like successful treatment. Interestingly, Akter et al. [23] has already reported that cfDNA in blood will be an ability screening marker for figuring out parasite variety in dogs.

In agreement with previously published data in particular in humans [34, 35], identity and acquiring sensitivity/ specificity of biomarkers is critical to be covered with inside the listing of exams which required to adjust to the treatment of patients, and therefore the initial diagnosis and selection to start remedy is also based totally at the results of the cfDNA index, so ROC curve evaluation became finished and located out maximum accuracy AUC of ROC for Baseline-third week 98.67% which gave a corresponding 100% sensitivity and 93.33% specificity. On the opposite hand, in line with the outcomes of the present study and based totally on the cut-off value, we recommend that dogs with distant TVT are probably discriminated from other puppies with an excessive percent of sensitivity while the usage of Line-99 primers for qPCR evaluation. Of course, similar research is desired to check this phenomenon. Additionally, evaluation of cfDNA integrity results confirmed that the amount of this index decrease than 0.3485 suggests the case has an ailment or confirmed a loss of a successful treatment for the duration of the weeks and it is able to be vital in clinician decisions. A preceding study mentioned that the cutoff point for cfDNA integrity in canine mammary tumors becomes 0.62 [21].

Lysyl oxidase (LOX) may be a cell-secreted amine oxidase which differentially regulated by status of disease or cancers [36] and plays a pivotal role in cancer progression, including metastasis, and is therefore is an attractive therapeutic target [15]. It’s been used differently in animals and has also been reported in cardiovascular disease of dogs [37] or copper deficiency in cattle [38]. Results of this study, investigated effects of treatment with Vincristine on LOX secretion by tumor cells which could promote treatment resistance. a major increase in lysyl oxidase activity was observed within the second and third weeks after treatment, which investigated LOX secretion by tumor cells which could promote treatment resistance [39]. Studies have shown that LOX leading to cancer niches where tumors can develop and metastasize. In other hand it has been shown to have an inhibitory effect in the development of cancer tumors [40, 41]. Pervious study for characterizing time0dependently of LOX activity showed that Type I Collagen and lysyl oxidase mRNA expression peaked in samples collected after 14 days of study [42]. Due to our results, LOX activity start increase after 7 days of first dose of vincristine, which because of histological changes to mediate crosslinking of collagen and elastin [43].

Unlike cfDNA, the quantity of enzyme activity within the first weeks is helpful in helping to diagnose or evaluate the response to treatment, in order that within the first week, with 60% sensitivity and 70% specificity, it can substrate animals that haven't received treatment with treated animals. within the comparison second week with baseline (100% sensitivity, 90% specificity and AUC 100%), we found that if the LOX activity is a smaller amount than 0.8648, it are often concluded that the treated animals don't respond well to vincristine and may be dosed or variety of treatment should be reviewed by a veterinarian. within the study of Saleem et al. (2019), the results of LOX evaluation with a high percentage for the choice of this enzyme in breast cancer were. Their report about LOX expression in canine mammary tumors (CMT), revealed that lysyl oxidase expressed as His-tagged fusion protein in prokaryotic expression vector was used for detection of circulating protein LOX in serum of CMT subjects. Their ROC results showed high sensitivity (90%) and specificity (85%) with histopathology because the reference standard and that they proposed LOX as a diagnostic biomarker and a putative prognostic candidate in CMT cases [28]. Examination of the relationship between cfDNA index value and enzyme activity showed that only baseline results had a major difference between the 2 markers if the 95% significance level is taken into account.

## Conclusion

The results of this study demonstrate which case we discover that these two markers had significant difference (Inverse relationship) which could help in quickly diagnose of TVT cases together with cytology. These markers within the diagnosis of TVT with using as liquid biopsy which might change in diagnosis of suspected patients in early-stages. Further studies should be completed in tumor subtype or other treatments to raised test a prognostic role and to verify applicability of thresholds of cfDNA integrity or LOX activities values.

## Materials and methods

### Samples

The 60 samples were specifically obtained and followed from 15 male dogs (range 5–12 years) in Iran between Jun 2020 and January 2021 which intravenously administered vincristine sulfate at a dose of 0.7 mg/m^2^ of body area [44]. Samples were obtained on the day of admission immediately before chemotherapy. Blood was drawn into EDTA-containing tubes and centrifuged at 1000 g for 10 min at 4 °C. Plasma was carefully removed, leaving 3–5 mm above the buffy coat, added to a tube and re-centrifuged at 1000 g for 10 min at 4 °C. Plasma was carefully removed and stored at -80 °C in 1 ml aliquots for cfDNA assay.

Exclusion criteria were a history of current disease, abnormalities detected on routine clinical examination, or significant abnormalities on a complete blood count.

### Extraction of circulating free nucleic acids and QC checks

For cfDNA analysis, DNA isolated from 0.5 ml citrated plasma using silica membrane-based DNA purification spin columns according to the manufacturer’s protocol (DNP™ Kit High yield DNA Purification Kit, CinnaGen, Iran). Concentrations of cfDNA on purified samples were measured in duplicate with UV absorbance at 260 nm employing a UV-spectrophotometer (JENWAY, UK). Real-time PCR was performed using YTA SYBR Green qPCR MasterMix2x. Briefly, the reaction was performed in 20 μL reaction volumes containing Master Mix, 0.5 μl of every primer, and 1 μL of the sample. The real-time PCR conditions consisted of an initial denaturation step for five minutes at 95 °C followed by 40 cycles for 15 s at 95 °C and annealing/extension for 1 min at 60 °C. A negative control (without the template) was performed on each plate. The sequences for RT-PCR primers were as follows: Line-99 forward, 5′-AAATGCAATGAAACGCCGGG-3′; Line-218 forward, 5′-TGGGAATGTGAACTGGTGCA-3′ and reverse 5′-TCTTTCGTTGGACACCGAGG-3′ [21]. Serial dilutions of (from 1–10,000 ng/mL) of genomic DNA obtained from the peripheral blood leukocytes of a healthy dog were analyzed, and also the resulting standard curves were wont to calculate the DNA concentrations of cfDNA in each sample. All samples were evaluated in triplicate, and a negative control (without template) was included in each plate [21].

### Lysyl oxidase activity

We comprehensively analyzed lysyl oxidase activity to measure LOX related to the disease. Blood samples were obtained on the day of admission immediately before chemotherapy in TVTs patients in sterile tubes, centrifuged at 2000 × RPM for 10 min, aliquoted into 1.5-mL tubes, and sera were preserved at − 80 ℃ until analysis [15]. Serum samples were diluted with an equal volume of phosphate-buffered saline and duplicate diluted samples were assayed for serum LOX concentration using a commercial kit (Kiazist Co, Iran). Briefly, 20 μL of the undiluted sample, 250 μL of the LOX Substrate Buffer, 5 μL of the horseradish peroxidase, and 1 μL of the LOX Probe was added to each well for every 5 samples and incubated for 1 h at room temperature. Then after adding LOX Lysis, the absorbance’s of samples and standards at 570 nm were measured immediately using Plate Readers (Biotech Instruments, INC, USA).

### Statistical analysis

Statistical analyses were disbursed using GraphPad Prism software version 9.0. Comparisons between every week for cfDNA and LOX data were analyzed by the t-test. For comparisons among over two continuous variables, an ANOVA test was performed. Pearson's correlation coefficient analysis was accustomed assess bivariate correlations between all the variables. The receiver operator characteristics (ROC) plot was obtained by calculating the sensitivity and specificity for each distinct observed data value and plotting sensitivity against 100%-specificity. The ROC curve was used to evaluate the optimal cut-off values for mortality prediction. A cutoff point on the curves was chosen to achieve the best compromise between sensitivity and specificity for fatal outcome.

## Supplementary Information

**Additional file 1.**

## Data Availability

All data generated or analysed during this study are included in this published article and its supplementary information file (with RAW DATA name file).

## Abbreviations

TVT: Transmissible venereal tumors
CTVT: Canine Transmissible venereal tumors
cfDNA: Cell-free DNA
LOX: Lysyl Oxidase
ROC: Receiver operator characteristics
ANOVA: Analysis of variance
